# Does GLUT4 Queue? A Mechanistic Mathematical Model for Insulin Response in Adipocytes

**DOI:** 10.1007/s11538-025-01490-6

**Published:** 2025-09-02

**Authors:** Brock D. Sherlock, Marko A. A. Boon, Maria Vlasiou, Adelle C. F. Coster

**Affiliations:** 1 School of Mathematics & Statistics, University of New South Wales, Sydney, 2052 NSW Australia; 2 Department of Mathematics and Computer Science, Eindhoven University of Technology, P.O. Box 513, Eindhoven, 5600 MB The Netherlands; 3 Faculty of Electrical Engineering, Mathematics and Computer Science, University of Twente, P.O. Box 217, Enschede, 7500 AE The Netherlands

## Abstract

Mammalian cells regulate their glucose levels by redistributing glucose transporter proteins within the cell. Glucose Transporter 4 (GLUT4) is the main insulin-regulated glucose transporter in mammalian cells. Insulin signals the redistribution of GLUT4 from intracellular compartments to the cell surface. The mechanisms of the release of GLUT4 and subsequent transport to the plasma membrane remain an open question. Here, a biologically plausible model of GLUT4 translocation is presented. Using a stochastic queuing model, we find that changing only the number of fusion sites available for GLUT4-containing vesicles as a function of insulin is sufficient to explain experimental observations. Thus, the activity of the fusion sites could be the primary determinant of the dynamics of GLUT4.

**Supplementary Information:**

The online version contains supplementary material available at 10.1007/s11538-025-01490-6.

## Introduction

Glucose transporter 4 (GLUT4) is a membrane-embedded passive transporter protein that facilitates glucose uptake in adipocytes (mammalian fat cells). The uptake rate is controlled by the number of GLUT4 proteins at the outer cell plasma membrane. GLUT4 is continuously recycled throughout the cell (Stöckli et al. 2011) and the spatial distribution of GLUT4 in the cell is regulated by insulin (Lizunov et al. 2012, 2013; Fazakerley et al. 2022). In the absence of insulin, GLUT4 is found mainly in intracellular membranes. However, in the presence of insulin, GLUT4 exocytosis increases and higher levels of GLUT4 are expressed on the plasma membrane.

Mean-field models propose two pathways for GLUT4 exocytosis: a recycling pathway, and a sequestration pathway (Brewer et al. 2014; Coster et al. 2004; Fazakerley et al. 2010; Xiong et al. 2010; Klip et al. 2019; Ray et al. 2023). The sequestration pathway removes GLUT4 storage vesicles (GSVs) from the recycling pathway, slowing (or halting) their transit from intracellular storage to the plasma membrane.

Dominant processes that act to control the dynamic balance of cellular GLUT4 have been identified using mean-field models (Brewer et al. 2016a, b, 2014; Coster et al. 2004; Fazakerley et al. 2010; Govers et al. 2004; Holman et al. 1994). For example, modelling and experimentation have identified that the main insulin-induced change is the increase in the exocytosis rate in adipocytes (Satoh et al. 1993; Yang and Holman 1993; Karylowski et al. 2004; Brewer et al. 2014, 2016a) while the endocytosis rate is largely insensitive to insulin levels (Brewer et al. 2014; Habtemichael et al. 2011).

The increase in the exocytosis rate could be attributed to the release of sequestered GLUT4 into the recycling pathway with a fast transit to the plasma membrane. The sequestration of GSVs is thought to occur in an insulin-dose-dependent manner (Coster et al. 2004; Govers et al. 2004; Habtemichael et al. 2011). The release of the sequestered GSVs causes a flush of fusion events to the plasma membrane (Stöckli et al. 2011; Satoh et al. 1993; Holman et al. 1994; Burchfield et al. 2013). Mean-field models support the sequestration hypothesis (Brewer et al. 2014; Coster et al. 2004), but do not elucidate what underlying mechanisms act on the system to elicit changes.

Here, we hypothesise that making the activity of the fusion sites insulin-dependent is in and of itself sufficient to explain the behaviour of the system in response to insulin. An increase in fusion site activity would increase the level at the cell surface even if all other rates remain constant. Inactive fusion sites could be a possible mechanism for sequestering GLUT4: it would be queued on microtubules, waiting to be released into the recycling pathway. The application of insulin could then activate some fusion sites releasing the sequestered GLUT4.

To test whether insulin acting only on the activity of fusion sites is sufficient to explain the experimental observations, we have developed a stochastic model, a closed queuing network, to model GLUT4 translocation; for more details, see Section 4. We model packets of GLUT4 as customers in the network and track their movement throughout cellular locations, identified through experimental observation (endosome store, microtubules, fusion sites, and the plasma membrane), modelled as service stations. The service rate at each station models the transit time through each location. We hypothesise that these rates are independent of insulin levels. We show that it suffices for only the number of fusion sites that work to have insulin dependence to explain the experimental observations.

Our hypothesised mechanism is informed by biological observations and dynamics apparent in experimental data sets, highlighted in Sections 2 and 3. From these observations, we develop a closed queuing model, consisting of only the necessary biological structures, to explain the system dynamics; see Section 4 for details. We test the efficacy of our model in Section 5 by fitting model parameters to experimental data of GLUT4 dynamics under differing insulin levels. We constrain the fit to allow insulin to act only on the activity of the fusion sites. By capturing the system dynamics with our constrained fit, we demonstrate that insulin action at the fusion sites can explain the dominant features of the observations.

## Biological Background

The current investigation focuses on the insulin-driven movement of GLUT4 in mammalian adipocytes (fat cells). In this study, we consider only the short-term insulin response of cells; i.e., we do not consider the creation and degradation of GLUT4 molecules. The response to insulin of already formed GLUT4 is of the order of minutes, while newly synthesized GLUT4 does not show significant insulin response for six to nine hours (Watson et al. 2004). It has also been suggested that the initial acute action of insulin is targeted to GLUT4-containing vesicles already near the cell periphery. This insulin-sensitive pool by the cell periphery is replenished by long-range transport from the perinuclear region, but the process takes somewhere of the order of 24 hours (Semiz 2003).

### Cell Structures

GLUT4 is a passive transporter protein that allows glucose molecules to cross the plasma membrane, driven by differences in glucose concentration. The rate of glucose transport into (and out of) fat and muscle cells is governed by the concentration of GLUT4 at the membrane and the duration for which these proteins are maintained at this location.

GLUT4 transport is facilitated by vesicles, which are packets of membrane that transport cargo, including GLUT4, throughout the cell and are destroyed and reformed in the process of fusing and budding from cellular membranes. GLUT4 is removed from the plasma membrane by a process in which a part of the membrane is budded off to create a vesicle. The GLUT4 embedded in the part of the membrane that forms the vesicle is carried away into the cell cytoplasm. After leaving the plasma membrane, the vesicles travel through the cytoplasm to the endosomes.

Endosomes are internal membrane structures. Endosomes are a key component in the recycling pathway: GLUT4 containing vesicles both fuse to and are formed from the endosomes. Vesicles formed at the endosomes can be recycled to the plasma membrane.

GLUT4 is also found associated with microtubules. Microtubules form a dense network of tracks within the cell, connecting regions near the inner endosomal membrane structures to the underside of the outer plasma membrane. There are many microtubules running in parallel between sites. Vesicles can be coupled to microtubules through molecular motors that transport them along the microtubule, for instance from the endosomes to the plasma membrane (Semiz 2003; Bose et al. 2002). The speed of the molecular motors has been observed to be independent of insulin, although insulin does increase the observed frequencies of movement (Semiz 2003). Microtubules may be a key component of the GLUT4 recycling pathway (Semiz 2003; Guilherme et al. 2000; Emoto et al. 2001; Molero et al. 2001; Fletcher et al. 2000; Olson et al. 2001). GLUT4 vesicles have previously been suggested to queue for fusion and transit along microtubules with the amount of queuing dependent on the extent of release of GLUT4 at the fusion sites (Koumanov et al. 2005). In this scenario, microtubules may have a permissive role, rather than a regulating role, facilitating access of vesicles to the fusion sites at the plasma membrane (Koumanov et al. 2005; Bose et al. 2004; Lizunov et al. 2005). Other studies have identified direct effects of insulin stimulation on microtubules such as the polymerization of microtubules, and an increase in micrtoubule density and curvature close to the plasma membrane (Batty and Langlais 2021; Dawicki-Mckenna et al. 2012; Olson et al. 2003).

When molecular motors have transported the vesicle, via microtubules, to the underside of the plasma membrane, the fusion site proteins (SNARE complexes) tether, dock, and fuse the vesicle membrane to the plasma membrane, delivering the vesicular GLUT4 to the cell surface (Dawicki-Mckenna et al. 2012). The association of a microtubule with a fusion site closely follows one hypothesis that microtubules are involved in the delivery, localisation, or scaffolding of signalling components or fusion machinery; see for instance (Eyster et al. 2006; Khandani et al. 2007). Similar positions on the cell membrane have been observed to be sites of repeated fusion events (Yuan et al. 2015; Karylowski et al. 2004; Mele et al. 2009a, b; Dawicki-Mckenna et al. 2012; Burchfield et al. 2013), and these sites are co-located with microtubules (Yuan et al. 2015; Mele et al. 2009a, b; Dawicki-Mckenna et al. 2012; Burchfield et al. 2013). For this study, we refer to sites of repeated fusion events as the *fusion sites*.

### Effects and Mechanisms of Insulin on GLUT4 Translocation

The concentration of GLUT4 at the plasma membrane is regulated by insulin, i.e., insulin governs the dynamic balance of GLUT4 exocytosis and endocytosis. GLUT4 is continuously recycled to and from the plasma membrane and internal membrane structures to regulate glucose uptake. GLUT4 is believed to be recycled to the outer plasma membrane via the endosomes (Fazakerley et al. 2022) but is also sequestered in storage compartments (Brewer et al. 2011). In the basal (no insulin) state GLUT4 is expressed at low levels at the plasma membrane and recycles both directly from the endosomes to the plasma membrane and as vesicles along microtubules to the plasma membrane (Mele et al. 2009b; Karylowski et al. 2004; Molero et al. 2001). When insulin is applied an increase in vesicular traffic is observed. Sequestered GLUT4 is released into the recycling pathway causing a rapid translocation of GLUT4 to the plasma membrane in an insulin-dose dependent manner (Coster et al. 2004; Govers et al. 2004, 2008; Habtemichael et al. 2011; Lopez et al. 2009).

Immunofluorescence can be used to observe fusion events and GLUT4 close to the cell membrane using techniques such as total internal reflection fluorescence (TIRF) microscopy. Two distinct stages of GLUT4 response to the application of insulin to the cell have been observed: an initial phase of rapid translocation of GLUT4 to the cell surface, followed by a ‘steady-state’ phase lasting for hours where elevated levels of GLUT4 are maintained at the plasma membrane (Fazakerley et al. 2022). In the first phase, within approximately three minutes, 50% of (the total cellular) GLUT4 is delivered to the surface in a flush of fusion events (Satoh et al. 1993; Holman et al. 1994; Stöckli et al. 2011). After the initial burst, the fusion rate returns to a steady lower level; in some cases it is similar to that observed in the basal case (Stöckli et al. 2011), or a heightened level still lower than the burst rate (Burchfield et al. 2013; Stenkula et al. 2010). Note that this burst refers only to the fusion event occurrence, the amount of GLUT4 at the plasma membrane increases and maintains heightened levels in the insulin stimulated state.

Increases in insulin have been observed to correlate with increased levels of GLUT4 transiting the plasma membrane; that is, there appears to be an increase in the pool of GLUT4 available for recycling (Govers et al. 2004; Brewer et al. 2014; Xiong et al. 2010; Klip et al. 2019; Ray et al. 2023). In Govers et al. (2004), it was hypothesised that a certain amount of GLUT4 was sequestered from the recycling pathway, particularly at low insulin levels, with the amount recycling increasing with increasing insulin level, which was also supported by other studies (Brewer et al. 2014; Romenskaia et al. 2025). Other authors (Xiong et al. 2010; Klip et al. 2019; Ray et al. 2023) hypothesise that GLUT4 is dynamically retained internally as a function of insulin, but also find an increasing rate of GLUT4 transport to the plasma membrane with increasing levels of insulin.

Multiple pathways influenced by insulin action have been identified. Here, we outline possible sites of insulin action that may have dominant effects on GLUT4 translocation. One possible site of insulin action is the microtubules where the molecular motor, mysosin Myo1c, functions in an insulin-dependent manner (Bose et al. 2002; Dawicki-Mckenna et al. 2012; Semiz 2003; Olson et al. 2003). However, depolymerisation of microtubules only partially impairs GLUT4 recycling and insulin-mediated GLUT4 translocation (Semiz 2003; Guilherme et al. 2000; Emoto et al. 2001; Molero et al. 2001; Dawicki-Mckenna et al. 2012; Karylowski et al. 2004; Huang et al. 2005; Olson et al. 2003) by about 40% (Fletcher et al. 2000). As disruption of the microtubules is unable to completely halt GLUT4 translocation, it has been hypothesised that the initial acute action of insulin is targeted to GLUT4-containing vesicles already near the cell periphery (Semiz 2003). Insulin has also been observed to increase the frequency of GLUT4 movement along microtubules, but the velocity of GLUT4 along the microtubules is not affected (Semiz 2003).

Various observations are in the literature to support insulin action at the fusion sites. For example, insulin stimulation has been observed to cause an increase in syntaxin4-containing SNARE complex formation in adipocytes, i.e., an increase in available fusion sites (Kioumourtzoglou et al. 2014). In Bai et al. (2007), the authors suggest that a key insulin-regulated step is the step after docking, a priming for fusion event. There is also evidence that insulin stimulates GLUT4 fusion, rather than the tethering of GLUT4 containing vesicles, in muscle cells (Lizunov et al. 2012).

Most mean-field models consider only basal and maximal insulin and do not consider a mechanism by which GLUT4 may be withheld from recycling at sub-maximal insulin levels. An exception to this is Fazakerley et al. (2022) where a retention-catalyst (such as a vesicle coat protein or a retaining Rab protein) has been proposed as a mechanism of GLUT4 sequestration. This was proposed as an extension of the three-compartment model in Holman et al. (1994) with the availability of the catalyst creating a finite capacity for the sequestered GLUT4 compartment. In the model of Fazakerley et al. (2022), insulin releases GLUT4 from the retention-catalyst causing a burst of fusions at the plasma membrane and the release of the catalyst to enable sequestration. This creates a one-out one-in type dynamic for GLUT4 in a sequestered compartment. The retention-catalyst model would require the amount of catalyst to be insulin-dependent in order to have differential sequestration.

The aim of this paper is to test a mechanistic hypothesis for insulin-regulated GLUT4 translocation consistent with both maximum and sub-maximal insulin responses. The literature identifies effects of insulin at both the microtubules and the fusion sites. Here we propose the dominant action occurs at the fusion sites. If insulin acts at the fusion sites, there are two possible actions to increase the rate of GLUT4 exocytosis: the service rate of the fusion sites is insulin-dependent, or the number of fusion sites active is insulin-dependent. Previous studies suggest that GLUT4 is sequestered away from the recycling pathway at sub-maximal insulin (Govers et al. 2004; Coster et al. 2004; Brewer et al. 2014; Romenskaia et al. 2025). There is experimental evidence for insulin impacting the number of fusion sites (Kioumourtzoglou et al. 2014) and how this may be enacted (Eyster et al. 2006; Khandani et al. 2007). In the following section we present quantitative data that will be used to assess the ability of our model to capture the system dynamics.

## Experimental Data

**Fig. 1:**
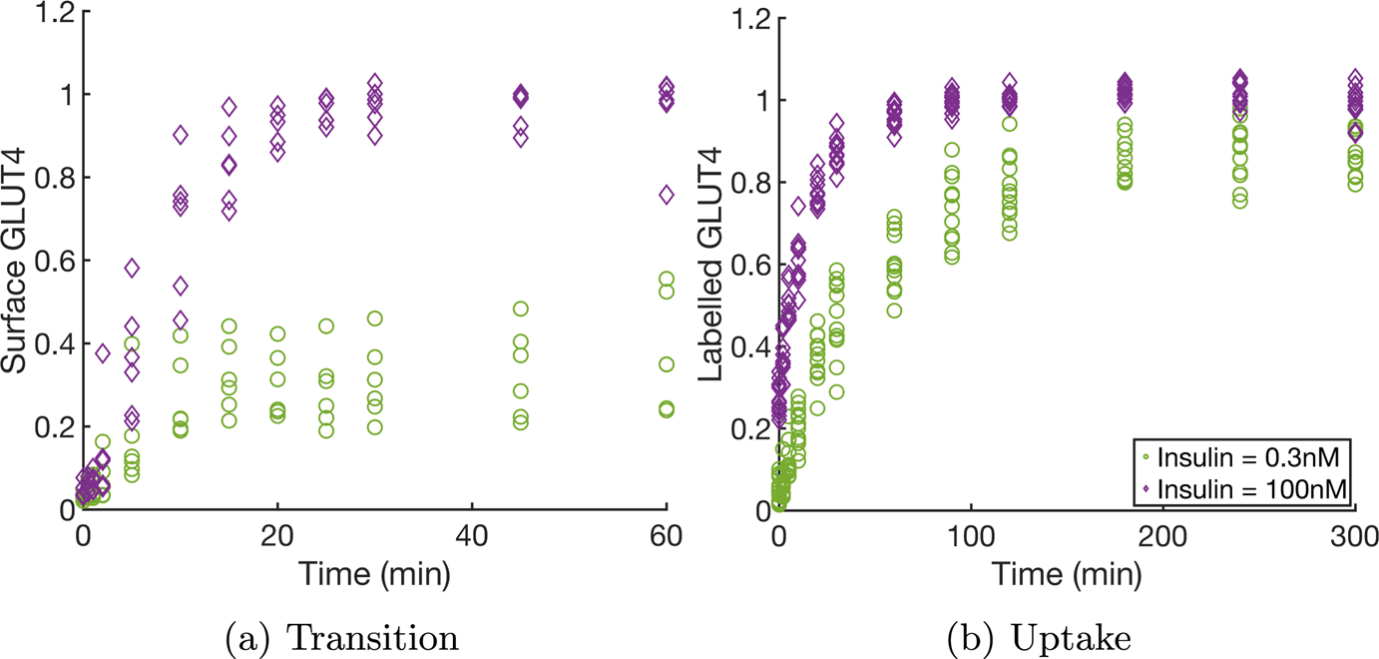
Example experimental data for a) transition and b) uptake experiments for two (out of nine) insulin levels: 0.3nM (green circles) and 100nM (purple diamonds) from Romenskaia et al. (2025). The transition data shows surface GLUT4 as a function of time, normalised to the average value at 60 minutes in 100nM insulin. The uptake data shows the level of GLUT4 that has transited the plasma membrane as a function of time, normalised to the average value at 300 minutes in 100nM insulin

To inform and test our model for GLUT4 translocation, we consider two experimental protocols: transition and uptake experiments. Data from these experiments (Romenskaia et al. 2025) will be used to constrain the parameter estimates for the model. Each experiment was repeated for up to nine insulin levels ranging from 0nM to 100nM; a sample of the data for two of the nine insulin levels is shown in Figure 1.

Each experimental protocol consists of bulk measurements, that is, each measurement is the fluorescence of many cells in a 96-well plate. Thus, the results are the average fluorescence over many thousands of cells. All data points are recorded as a proportion of the maximum fluorescence; i.e., all data points, for all insulin levels, are normalised to the mean steady-state value (60 minutes for transition and 300 minutes for uptake) in 100nM of insulin.

Measurements of the system are taken at predetermined sample times to create a time-series dataset. Each measurement is taken from a different well of the 96-well plate, destroying the cells in that well in the process. That is, each data point is taken from an independent group of cells, so it is not possible to observe the longitudinal dynamics of the same cells due to the destructive measurements.

***Transition Experiment***

The transition experiment tracks the amount of GLUT4 that is at the plasma membrane as a function of time. The cells are initially in steady state with a set amount of insulin (usually basal, i.e., zero insulin). At the start of the experiment, a fixed level of insulin is applied and maintained. Note that the initial insulin level is always less than or equal to the final concentration of insulin, as decreasing the insulin level is not possible in these experiments. Different wells of cells are exposed to insulin for each of the protocol sample times and then put on ice (stopping translocation processes), the surface GLUT4 is tagged and the fluorescence measured. In this way, the experiment records the population of GLUT4 at the plasma membrane as a function of time, as the system transitions from the steady state in the initial (usually basal) level of insulin to the steady state in the presence of the applied insulin dose. In the experiments (Romenskaia et al. 2025), each measurement was normalised to the steady state level at maximal insulin (100nM), where 60 minutes after insulin application was determined to be sufficient to reach the new steady state. Data for the transition from basal to one of four insulin levels: 0.3, 3, 30, and 100nM was obtained with a minimum of six repeats at each of 0, 0.5, 1, 2, 5, 10, 15, 20, 25, 30, 45, and 60 minutes.

***Uptake Experiment***

In the uptake experiment, the cells are initially in steady state in the presence of a set amount of insulin (possibly 0nM). The experiment permanently immunofluorescently tags the GLUT4 molecules as they transit to the plasma membrane. Different wells of cells are then put on ice, stopping any translocation, at predetermined sample times, and the total fluorescence is measured. The fluorescence is a proxy for the amount of GLUT4 that has transited the plasma membrane since the start of the experiment to the time of measurement. Initially, the measurement is the amount of GLUT4 at the plasma membrane and this increases with time as the unlabelled GLUT4 molecules transit to the plasma membrane. The amount of transiting GLUT4 levels out with time, the final measurements being the total amount of GLUT4 recycling in the presence of the particular insulin level. The data at different insulin doses in Romenskaia et al. (2025) was normalised to the amount recycling in maximal insulin (100nM) at 300 minutes. Data for the uptake protocol was obtained for nine insulin levels: 0, 0.03, 0.1, 0.3, 1, 3, 10, 30, and 100nM with a minimum of ten repeats at each of 0, 2, 5, 10, 20, 30, 60, 90, 120, 180, 240, and 300 minutes.

## Queuing Model

Our modelling choices were informed by observations from the biological background in Section 2 and the observed dynamics in the experimental data in Section 3, that is, microtubules form a key component of the translocation pathway, microtubules and fusion sites are co-located, and insulin impacts the operation of fusion sites.

We wish to explore the hypothesis that it is sufficient to restrict the effects of insulin to the activity of the fusion sites (with other processes unaffected) to replicate the experimental observations. A fusion site could be active or inactive with sites activating as an insulin dose response; the more insulin there is, the more sites are active and only in the active state a fusion site can tether and fuse vesicles to the plasma membrane. GLUT4 sequestration would then occur on the microtubules delivering the GLUT4 to inactive fusion sites, causing a build up along the microtubule. The subsequent activation of fusion sites with insulin could then release GLUT4 into the recycling pathway, while also increasing the rate of fusion events to the plasma membrane.

In the basal (no insulin) state GLUT4 is expressed at low levels at the plasma membrane and recycles both from the endosomes directly to the plasma membrane (Karylowski et al. 2004; Molero et al. 2001) and via vesicles travelling along microtubules to the plasma membrane (Semiz 2003; Guilherme et al. 2000; Emoto et al. 2001; Molero et al. 2001; Fletcher et al. 2000; Olson et al. 2001; Koumanov et al. 2005). For convenience and to keep the network architecture as simple as possible in the current model, the direct mechanism for basal recycling independent of microtubules is not explicitly modelled in this study. Rather, both basal recycling routes were combined and modelled as an always active route via the microtubules. This mechanism limits the rate that can be transported via that route. A separate explicit route from the endosomes to the plasma membrane could be added in a future expanded model with additional parameters.

We model the system with a closed queuing network that allows us to simulate the dynamics observed from the experiments. A queuing network consists of customers and stations. Each station is a queue with one or multiple servers. Customers visit the stations and spend some amount of time, dependent on the characteristics of the station, waiting to complete service. At completion of service customers are routed to a new station. A ‘closed’ queuing network means the customer population is constant and is routed internally within the system; there are no external arrivals or departures. Queues have previously been used to describe biological systems such as inhalation toxicology (Wu 1998), glycolysis (Clement et al. 2020), Krebs cycle modelling (Kloska et al. 2021), and the insulin signalling pathway (Kloska et al. 2022; Jezewski et al. 2014). In the current investigation, a queuing model was implemented in which the only insulin-dependent parameter was the activity of the fusion sites, with the service times of the stations in the network being insulin-independent. It can be shown that allowing the service times to be insulin dependent rather than the fusion site activity can alter the plasma membrane level of GLUT4 in an insulin-dependent manner, but this configuration of the system does not enable the observed sequestration of GLUT4.

**Fig. 2:**
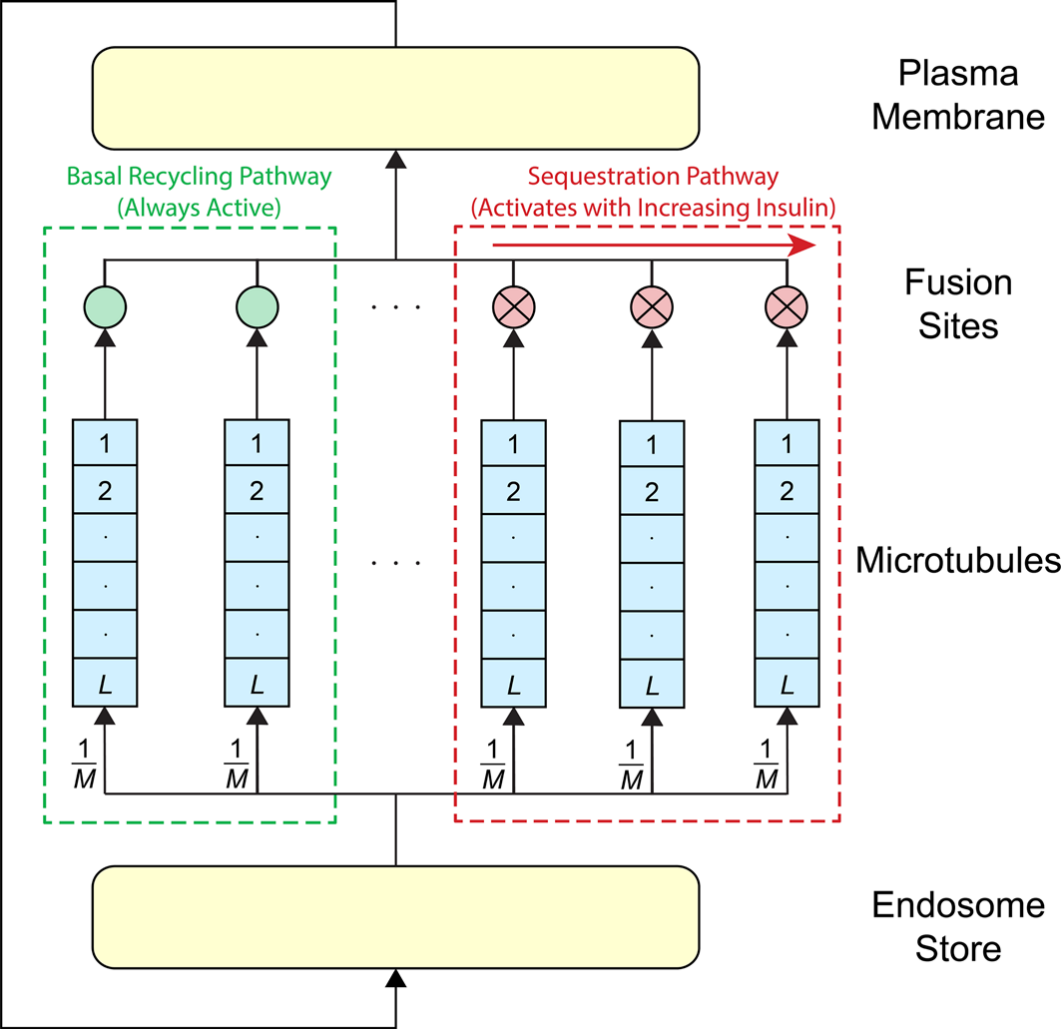
Schematic of the closed queuing network. *N* vesicles are routed cyclically through the endosome store, the *M* microtubules, the *M* associated fusion sites, and plasma membrane. Arrows denote the direction of travel through the system. Split paths have the probability of a particular branch being chosen shown, i.e., a microtubule branch is chosen with probability $\frac{1}{M}$. The microtubules queues have capacity *L*, and the fusion sites are either active or inactive, set by *p*(*I*) (green for active, red with cross for inactive). GLUT4 is sequestered in microtubules associated with an inactive fusion site, as part of the sequestration pathway. Additional fusion sites activate and enter the recycling pathway with increasing insulin levels, *I*. There is a certain fraction of the fusion sites that are always active, allowing continuous recycling of GLUT4 as part of the basal (no insulin) recycling pathway

Our queuing model, Figure 2, consists of four stations, each correlating to a biological structure in the GLUT4 translocation pathway: the endosome store, the microtubules, the fusion sites, and the plasma membrane. These stations represent key stages in the microscale exocytosis of GLUT4 that have been identified through experimental observation (Mele et al. 2009b, a). This queuing system was used in our previous work (Sherlock et al. 2024) to determine appropriate measures to drive parameter inference in such stochastic systems.

In our model, GLUT4 moves sequentially through each of the four stations. GLUT4 in the model is assumed to be packeted into vesicles each with identical loading, although, in reality, there is heterogeneity in the amount of GLUT4 molecules carried by each vesicle (Stöckli et al. 2011). Each of these *N* vesicles is a customer in the queuing network and vesicle trafficking is used as a proxy for GLUT4 movement.

Although vesicles are destroyed and reformed at the plasma membrane and endosomes, the model in effect leaves the GLUT4 at these stations packeted into vesicles. In reality, when vesicles fuse to these membranes, the vesicle membrane is incorporated into the main membrane surface and the embedded GLUT4 can then diffuse laterally. New vesicles can then be formed, budding off parts of the main membrane (including any embedded proteins). In the model, we assume that these processes occur in a one-to-one manner such that the total number of vesicles in the system remains constant. The reformed vesicles are also assumed to have the same size and loading as the other vesicles in the system. The time taken for the diffusion of GLUT4 in the membranes and the reformation of vesicles is incorporated into the service time at each of these membrane stations.

We consider all switch times to be zero; that is, the time to move between stations is zero, as this movement time is modelled as part of the service rate at each station. All service times are assumed to be exponential.

***Endosome Store***

The storage of GLUT4 in the endosomes, or endosome store, is represented by an infinite-server queue as we assume it has sufficient capacity to hold all vesicles at any time. The service time is exponentially distributed with rate $\mu _S$. It encapsulates the time the vesicle is stored in the endosome store (incorporating the diffusion of GLUT4 and packeting into vesicles) and the time for the vesicle to bud off, travelling to, and binding with a molecular motor at the microtubules. Vesicle customers, at completion of service, either move to the microtubules, or are blocked if the chosen microtubule is at capacity. Blocked vesicles re-enter service at the endosome store and a new service time is drawn from the same exponential distribution.

***Microtubules***

The microtubules consist of *M* parallel multi-server queues. Each of the queues consists of *L* parallel, zero-buffer servers, i.e., each microtubule can hold up to *L* vesicles. A vesicle chooses a queue with probability $\frac{1}{M}$. If the chosen microtubule is at capacity, i.e., all *L* servers are occupied, the customer is blocked, returned, and re-serviced in the endosome store. At completion of re-service at the endosome store, the vesicle attempts to re-enter the microtubule station via the same mechanism; the failed attempt does not change the probability of entering the queues.

The microtubules have finite length (about $10 \mu m$ (Semiz 2003)) so it is reasonable to assume that some finite number of vesicles are able to occupy the microtubule at any time, proportional to the size of the vesicles. For the model, we assume that vesicles must attach and detach at the ends of a microtubule, that is, they cannot join a microtubule partway down the microtubule jumping ahead of other vesicles. We also assume the vesicles remain attached to the same microtubule as they transit its length. Thus, each of the *M* microtubule queues maintains first-in-first-out (FIFO) ordering so that vesicles must leave the queue in the same order of arrival, regardless of their server.

Upon arrival at the *m*th queue, the *i*th customer (in that queue) immediately enters service. We now define the service process for each microtubule. Let the arrival and departure times of the *i*th vesicle in the *m*th queue be denoted $A_{m,i}$ and $D_{m,i}$ respectively. The service time, $S_{m,i}$, is drawn from an exponential distribution with rate $\mu _{M}$. Biologically, the service time represents the time needed for a molecular motor to transport the vesicle the length of the microtubule, if unimpeded.

We define an ‘increment time’, $I_{m,i}$, which is randomly sampled from an exponential distribution with rate $(L)\cdot \mu _{M}$. The increment time is only used when a customer is blocked in the microtubules and needs to move to occupy the position at the head of the queue. Biologically, this models the extra time needed for a vesicle to transit to the very end of the microtubule to be unloaded; this cannot occur if another vesicle occupies this position. When the position at the head of the queue becomes vacant, the blocked vesicle can manoeuvre into this ‘unloading zone’.

Vesicles may also be subjected to a blocking time, $B_{m,i}$, if the next station, the associated fusion site, is at capacity or inactive in which case vesicles cannot be unloaded from the associated microtubule. The blocking time is zero if the following fusion site is empty and active. If the next station is at capacity or inactive, $B_{m,i}$ will account for the time a vesicle spends in the microtubule until it can be unloaded. In this case, we assume that the vesicle remains in the microtubule server, rendering it unavailable. The associated fusion site may never become active. In this case, we take $B_{m,i}$ to be infinity, blocking the customer (and all customers in the same microtubule with a later arrival time) indefinitely. It is important to observe that if one of the *L* servers in the queue becomes indefinitely blocked, all other servers (in that queue) become indefinitely blocked due to FIFO ordering. In this case, the microtubule will reach capacity and no customers will enter or leave this microtubule, sequestering customers from the recycling pathway.

As each microtubule is identical, the subscript *m* is dropped in the following equations for clarity; each equation applies to an individual queue; i.e., there is no interaction across different microtubules. To maintain the FIFO ordering of each queue, the departure time of each vesicle is dependent on the departure time of the previous arrival in the queue; that is

1 $$\begin{aligned} D_i = {\left\{ \begin{array}{ll} A_i + S_i + B_i, & \text {if } A_i + S_i \ge D_{i-1}, \\ D_{i-1} + I_i + B_i, & \text {if } A_i + S_i < D_{i-1}. \end{array}\right. } \end{aligned}$$

At the departure time, the customer moves to the next station, which is the fusion site associated with the microtubule. Note, $D_i, A_i, S_i, B_i$, and $I_i$ are directly dependent on the other parameters of the system and are determined as part of the discrete event simulation, with the equations above included for completeness of the description of the model.

***Fusion Sites***

The fusion sites are modelled by *M* parallel single-server, zero-buffer queues; each queue corresponds to a unique microtubule. That means vesicles leaving the *m*th microtubule are routed to the *m*th fusion site, entering service if the server is not blocked. Each fusion site has an exponentially distributed service time with rate $\mu _F$. The service time encapsulates the time it takes the fusion site to tether the vesicle, dock, and fuse the vesicle to the plasma membrane.

Fusion sites have the property of being active or inactive. They are active with probability *p*(*I*), where *I* is the insulin level. The state of the fusion sites is determined at the time of insulin application. Once they are in a given state, they do not change state unless the insulin level changes. If active, they behave as described above. However, if they are inactive, then they do not accept any customers and block the path to the plasma membrane. This prevents vesicles from departing the associated microtubule. If the server of the *m*th fusion site is occupied, the vesicle is blocked after service at the microtubule. Blocked vesicles are immediately transferred from the microtubule when the fusion server becomes available. At completion of service, customers are routed to the plasma membrane.

In line with the hypothesis that the fusion sites are the only insulin-dependent stage in the system, the active probabilities are considered to vary with the insulin level, and all other system parameters are constant across all insulin levels.

***Plasma Membrane***

From the fusion sites, vesicles are deposited to the plasma membrane, which is modelled as an infinite server queue as we assume it has sufficient capacity to hold all vesicles in the system. The service time at this station is the time that the GLUT4 remains on the surface, including also the time for a vesicle to reform and bud off from the membrane. The service times are exponentially distributed with rate $\mu _{P}$. After service at the plasma membrane, customers return to the endosome store and begin the cycle again. Recall that the switch times are taken to be zero. The transit time of the vesicle to the endosome is also included as part of the time spent on the membrane.

***Correspondence to Experiments***

The experimental assays deliver outputs that are the average of many thousands of cells at each time sample (with different cells for each sample). Here we assume that the dynamics of the cells in independent wells are representative of the evolution of a single cell. In this study, we compare repeated, but independent, instances of our stochastic model of a single cell to these repeated measurements of averaged independent cell dynamics.

Some of our model variables, such as queue lengths, correspond directly to experimental observations. We also create model outputs that represent other biologically observable measurements. Additionally, the greater observability in the model compared to what is possible experimentally also allows us to explore the underlying dynamics of the system.

The transition experiment tracks the amount of GLUT4 present at the plasma membrane as a function of time. This is equivalent to the queue length of the plasma membrane in our queuing network as a function of time after the state of the system changes due to a change in insulin. We normalise the queue length to the steady state queue length in maximal insulin in line with the experimental normalisation.

In the uptake experiment, the cells are in a steady state with respect to a fixed amount of insulin. It tracks the amount of GLUT4 that has transited to the plasma membrane – labelling it as it first cycles there as a function of time. The model implements this protocol by tagging unique vesicle visits to the plasma membrane. Again, the model output is normalised to the steady state in maximal insulin in line with the experiments, i.e., the number of vesicles as a proportion of the number tagged in maximal insulin.

## Parameter Optimisation

To assess the model, multiple instances were simulated. Model outputs were obtained via a discrete event simulation implemented using Java OpenJDK 19.0.1. The simulations were performed using the computational cluster, Katana, supported by Research Technology Services at UNSW Sydney. Each run was initialised with all vesicles in the endosome station and the parameters set to the basal state; i.e., fusion server activity determined by the active probability *p*(0). The simulation then ran for 500 simulation time units (the start up time) to allow the distribution of vesicles at each station to reach the first equilibrium, i.e., the basal steady state. The simulated transition experiment was then initiated by changing the system to the insulin level parameters; i.e., fusion server activities determined by the active probability *p*(*I*), where *I* was the applied insulin level. From the onset of the transition experiment, the simulation was allowed to run for 500 time units to allow the system to reach the second steady state (the insulin steady state) before the simulated insulin uptake experiment was initiated for the given insulin level.

The 13 sets of GLUT4 time-series data of Romenskaia et al. (2025), detailed in Section 3, were used to simultaneously optimise the model parameters. In this study, we chose to fix our network architecture, i.e., the number of microtubules, their length, and the number of vesicles. These are not expected to be of the same magnitude as a physical cell, but rather a scaled version in which we can explore the efficacy of our hypothesis of insulin action. We considered the network architecture to consist of the number of microtubules, fixed at $M = 450$, and the microtubule capacity, fixed at $L = 50$. All 13 experimental data sets constrained the service rates (endosome store service rate, $\mu _S$; microtubule service rate, $\mu _M$; fusion site service rate, $\mu _F$; and plasma membrane service rate, $\mu _P$) and each service rate was common to all experiments.

The active probability, *p*(*I*), changed between experiments at different insulin levels, but was the same for both the transition and the uptake experiment for that insulin level, i.e., nine active probabilities were set, one for each insulin level. In this way, four sets of two datasets (pairs of transition and uptake experiments observed at the same insulin level) each constrained an active probability (four in total). Five single datasets (uptake experiments where no transition experiment was undertaken for that insulin level) each individually constrained a further five active probabilities. The number of experiments which constrained each active probability is given in Table 1.

To optimise the model parameters, the model output was calculated for each parameter set in the search using a discrete event simulation. For each parameter set, 100 repeats of the model output were generated. Here, each ‘repeat’ refers to a single realisation of the model for every insulin level (0, 0.03, 0.1, 0.3, 1, 3, 10, 30, and 100nM) across the transition and uptake experiments. The distance measure described in Sherlock et al. (2024) was used as the objective function in our inference using a line search, with the 100 model repeats compared to the experimental data.

In our earlier work, we performed a preliminary study with the same model and synthetic data to determine a suitable distance to use to optimise the parameter values (Sherlock et al. 2024). Our previous study found that a hierarchical distance was suitable for parameter inference: a Wasserstein-1 distance of samples at each time point, accumulating the time-point distances into experiment distance by taking the mean across each experiment, and finally a combined distance by taking the L2-norm of the experiment distances. Here, this combined distance was then minimised using a line search algorithm (details given in Appendix A), to determine optimal parameter sets.

Line search, or gradient descent methods in general, have difficulty when many local minima are present or when the gradient is small (Gershenfeld and Gershenfeld 1999; Press 2007). To overcome the issue of local minima, a region surrounding the termination point of the line search was sampled on a grid to search for other minima. The region was defined as plus-minus 50% of the terminated parameter value for the service rates and the number of customers. The calculation of the distance, the line search, and visualization of the results were implemented in Matlab (Version R2023b, Mathworks 2023).

### Start Points and Tolerances

The active probabilities required to sequester a set number of vesicles can be calculated from the uptake data if the number of vesicles, *N*, number of microtubules, *M*, and capacity of the microtubules, *L*, are known. In order to retain variability in the model outputs in the maximum insulin stimulated steady state the maximum level of vesicles recycling in the model was set to be 90% of the vesicle population (with the remaining 10% remaining sequestered). Using the initial values for *N*, *M*, and *L*, we then determined the initial values of the active probabilities so that the sequestered population in the model matched the insulin stimulated steady states for the uptake experiments.

To determine a suitable start point for the service rates, the service rates were randomly sampled in the ranges given in Table 1. Note, the range of the microtubule rate is smaller than that of the other rates as we attempted to constrain this to a realistic value, i.e., we expect this rate to be slower than the fusion rate (Bai et al. 2007; Stenkula et al. 2010; Pampaloni et al. 2006). For this sampling, the number of vesicles was set at 25000 with the start values of the active probabilities given in Table 1. The parameter set resulting in the smallest distance was iteratively chosen and the space around this point sampled. The best point was then chosen as the start point for the algorithm. This approach was necessary to ensure a sufficiently good start point (Sherlock et al. 2024).

The start point used for the optimisation is given in Table 1. Our fitting algorithm used the standard step-size tolerance of $10^{-6}$ on parameter changes (i.e., the smallest parameter change before termination) for all parameters bar the number of vesicles, *N*, which had a minimum step-size of 1000 (the number of vesicles is orders of magnitude larger than the other parameters and is required to be an integer). A lower bound of 0.01 was used for all active probabilities, *p*(*I*). This lower bound avoided too few fusion sites being active, i.e., even in zero insulin some fusion sites are active to have GLUT4 recycling. The lower bound on the number of vesicles was 25000, to ensure there were non-sequestered customers in the system. The lower bound on all service rates was $10^{-6}$. An upper bound of 1 was used for all active probabilities and an upper bound of 100000 was used for the number of vesicles. No upper bound was set for the service rates. There was no termination for maximum function evaluations. All experiments and timepoints were weighted equally.

## Results

**Fig. 3:**
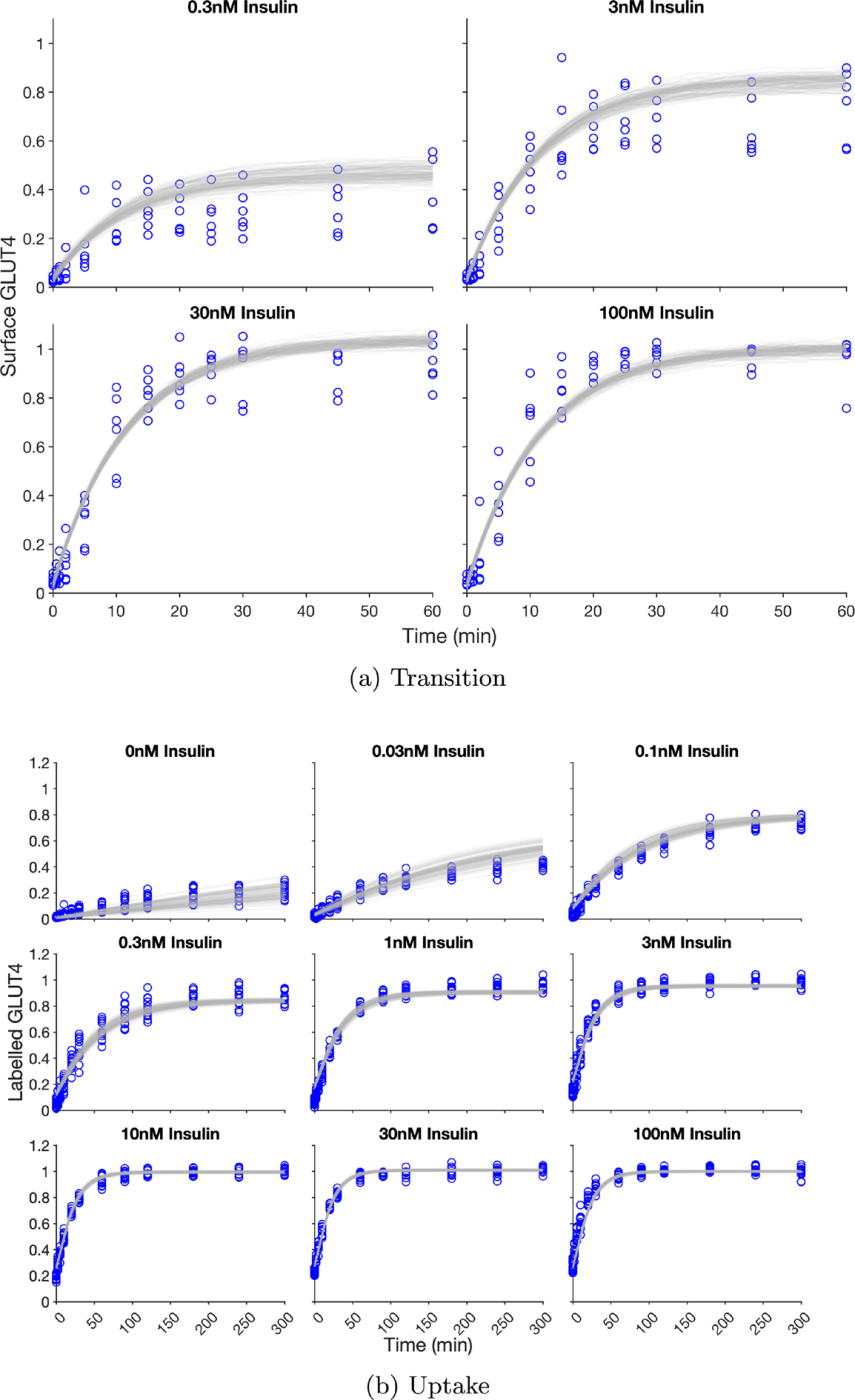
Correspondence between the optimised model and a) transition and b) uptake data, where the level of insulin is indicated above each plot. The experimental data is shown by blue circles. Traces for 100 realisations of the model outputs using fitted parameter values, given in Table 1, are shown by the grey lines. The traces sample the model output at half-minute increments. The transition data shows surface GLUT4 as a function of time, normalised to the average value at 60 minutes in 100nM insulin. The uptake data shows the level of GLUT4 that has transited the plasma membrane as a function of time, normalised to the average value at 300 minutes in 100nM insulin

To test whether our queuing model is a good description of the observed dynamics, we fitted the model to the experimental data outlined in Section 3. The parameter values obtained from fitting are shown in Table 1. In Figure 3, we show 100 realisations of the model output, with the optimised parameter values, for the transition and the uptake experiments. Good correspondence between the model output and the experimental data across both the transition and uptake experiments can be seen at all insulin levels. The model output predominantly lies within the range of the experimental data. The model overshoots the steady state in 0.03nM insulin and narrowly undershoots the steady state level in the upper end of the physiological range of insulin (1nM) for the uptake experiment; the good correspondence of earlier timepoints indicate the recycling rate is good but the model fails to recycle the correct amount of GLUT4. The variance of the model output better matches the variance of the experimental data in lower insulin levels for both the transition and uptake experiments. In higher levels of insulin the the model output exhibits much lower variance than the data.

**Table 1:** Datasets and model parameters. In the model fitting, the parameters were taken to be common to all insulin levels with the exception of the active probabilities. The available datasets for the transition and uptake experiments at different insulin levels are indicated by tick marks. Note that the parameters for insulin levels without both transition and uptake datasets were less constrained. Optimal values were obtained from the simultaneous fitting of experimental data sets of Romenskaia et al. (2025), see Section 5. Start points for the fitting algorithm and final fitted values are shown with ‘-’ indicating that the parameter was not included in the fitting algorithm. If the parameter was not including in fitting, it was set at the start point value

		Insulin Level (nM)								
		0	0.03	0.1	0.3	1	3	10	30	100
Transition					$\checkmark$		$\checkmark$		$\checkmark$	$\checkmark$
Uptake		$\checkmark$	$\checkmark$	$\checkmark$	$\checkmark$	$\checkmark$	$\checkmark$	$\checkmark$	$\checkmark$	$\checkmark$
Endosome Store Service Rate (customers/min)		$\mu _S$								
	Search Range	[0.01, 100]								
	Start Point	0.0957								
	Fit Value	0.2617								
Microtubule Service Rate (customers/min)		$\mu _M$								
	Search Range	[0.1, 10]								
	Start Point	0.2447								
	Fit Value	2.2459								
Fusion Site Service Rate (customers/min)		$\mu _F$								
	Search Range	[0.5, 400]								
	Start Point	1.0730								
	Fit Value	3.9483								
Plasma Membrane Service Rate (customers/min)		$\mu _P$								
	Search Range	[0.01, 100]								
	Start Point	0.1400								
	Fit Value	0.0869								
Active Probability		*p*(0)	*p*(0.01)	*p*(0.1)	*p*(0.3)	*p*(1)	*p*(3)	*p*(10)	*p*(30)	*p*(100)
	Search Range	[0.01, 1]								[0.8, 1]
	Start Point	0.1020	0.3034	0.6318	0.7646	0.8512	0.8812	0.8945	0.9015	0.8889
	Fit Value	0.0309	0.1123	0.2907	0.4235	0.6200	0.7700	0.8945	0.9416	0.9099
Number of Vesicles (per cell)		*N*								
	Search Range	[24000, 100000]								
	Start Point	25000								
	Fit Value	72000								
Number of Microtubules (per cell)		*M*								
	Search Range	–								
	Start Point	–								
	Fit Value	450								
Capacity of Microtubule (per microtubule)		*L*								
	Search Range	–								
	Start Point	–								
	Fit Value	50								

**Fig. 4:**
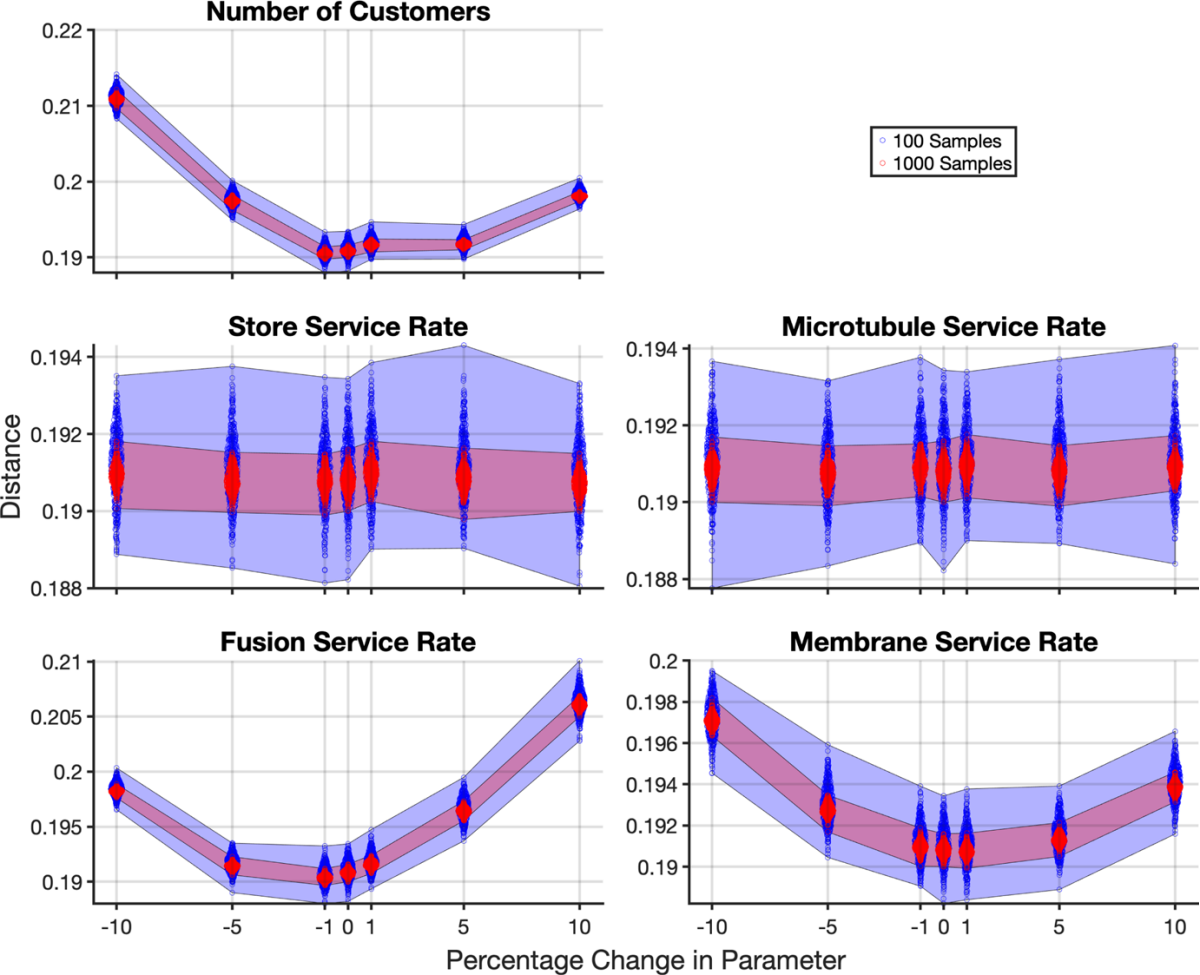
Combined distance as a function of the parameter values for 500 sets of model outputs with 100 model realisations (blue) and 1000 model realisations (red). All parameters were set to their optimal values, except the single parameter being altered. The plots show the distances from 500 sets of the given number of model realisations (where the width of the scatter at each parameter value indicates the distribution of the points). The blue and red regions show the maximum and minimum distances found from the sets of the 100 and 1000 model realisations respectively

To understand the influence of model parameters on model output and identify potential rate-limiting steps, a sensitivity analysis was performed. The change in distance between the model output and the data as each parameter was altered is shown in Figure 4. Here, each parameter was varied by plus and minus 1, 5, and 10 percent, with the other parameters fixed at their optimal value. It is clear in Figure 4, that the distance is relatively insensitive to changes in the microtubule and store service rates, while the fusion and membrane service rates, and the number of customers, *N*, are sensitive parameters.

It is interesting to note that the insensitive parameters are those internal to the cell, preceding the fusion sites. These are not observable experimentally. We found in the model with optimal parameters that the microtubules were almost always operating at capacity (data not shown). This indicates that the fusion sites are acting as a rate-limiting step, or bottleneck in the system. In this case, there is a high level of blocking in the network and increasing the service rates in stations behind the bottleneck (the store rate and microtubule rate) has no effect on the system.

The robustness of the distance measure as a function of model output sampling was investigated. As the model is stochastic, each model realisation (i.e., a single simulation run) is different. The distance is calculated by taking some number of model realisations to compare to the experimental observations. If the process is repeated, the set of model realisations is different, even if the same parameters are used. In Figure 4 we show the range of distances calculated for the same set of parameters when taking different sets of the same number of model realisations (sets of 100 and 1000 model realisations, each repeated 500 times). As the system is stochastic, different samples of the system result in a range of distances between the model and the experimental data, as also explored in Sherlock et al. (2024). In other words, rather than a single value for the distance for a given set of parameters we have a band of distance values. We determined that the range of values the distance function takes from different samples of the model outputs, which we will refer to as the distance noise, caused the optimisation algorithms to not always terminate at reproducible points; each time the optimisation was implemented a different minima (within some region) was found due to the different sampling of the model output.

The magnitude of the distance noise is dependent on both the parameter values and number of model realisations and samples taken of the model. This variability in the distance is illustrated in Figure 4 where a single parameter is altered from the optimal parameter set. Here it can also be seen that the Store and Microtubule service rates are largely insensitive – there is a large range of parameter values for these rates which produce indistinguishable distances, making it hard to identify single optimal parameter values. It can also be seen that the number of instances in the sample can affect the range of distances produced. This suggests that in surveying a parameter space there is a possibility that a ‘smaller’ distance can be found if the search is not comprehensive. This problem can in part be alleviated by increasing the number of realisations of the model, for example, to 1000 realisations, to minimise the range of distances returned for any given parameter set. However, the computational cost this incurs can become prohibitive for fitting; for example, to simulate a single sample with the optimal parameters for the transition and uptake experiments at each insulin level takes approximately 85 seconds in the current study.

**Fig. 5:**
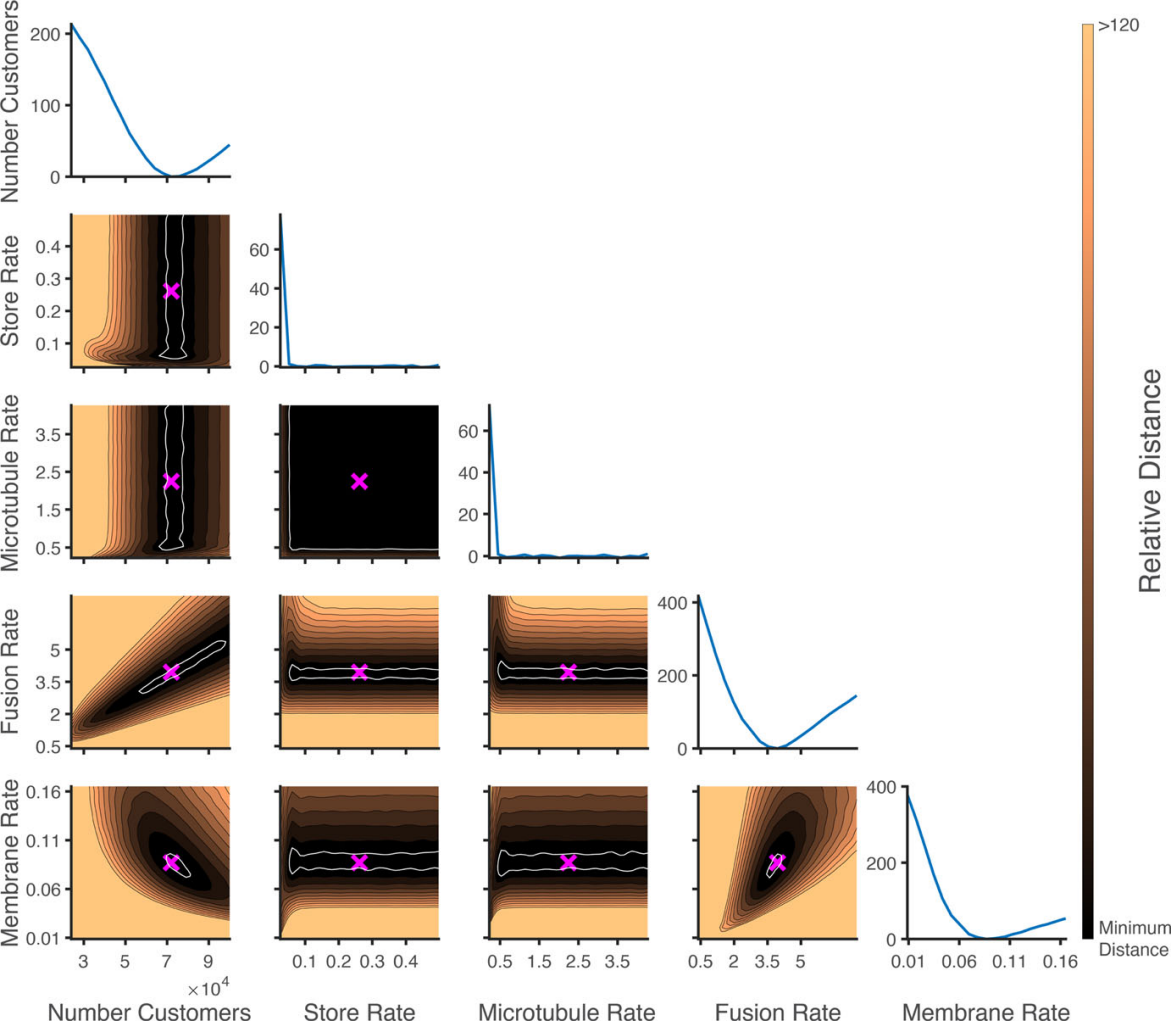
Contour maps of the distance as a function of the model parameters, using 100 model realisations. Each subplot varies two model parameters with all other parameters fixed to their optimal values. The optimal parameter values are marked by a magenta cross on each subplot. The colour indicates the percentage change in the distance relative to the optimal value. The white line indicates the contour for the uncertainty region, i.e., relative distances less than 2%. The diagonal plots show the distance as a function of a single parameter with all other parameters fixed at their optimal values

We found, however, that the distance landscape is steeper in regions further away from the optimal values, see for instance Figures 4 and 5. In this case, this allows for less overlap in the range of distances between nearby parameter sets. However, in regions close to the optimal parameter values (where the distance landscape was flatter) the distance noise had more impact. This demonstrates that the determination of an optimal parameter set is not just dependent on the parameter values but also on the inherent model output sampling error. To better navigate the flatter region of the distance landscape in this study, once the line search reached a clear valley in the distance landscape, the number of model samples was increased and the entire valley surveyed. This allowed the optimal parameter set to be refined. One approach for determining the optimal parameters is to define a minimum region, i.e., accept all parameter values that return distances under a threshold as ‘optimal’. This threshold can be set based on the banded regions of resampling errors shown in Figure 4.

Distance landscapes were calculated, using 100 realisations of the model for each parameter set, to visualise the fitting surface and ensure the optimal parameters are centred in some valley. Parameters were varied pairwise about the fitted values and the landscape of the distance is presented in Figure 5. Each of the four rate parameters (endosome store, microtubules, fusion sites, and plasma membrane) and the number of customers were varied in a range of plus and minus 90% around the fitted value. We extended the range to 90% as smaller regions did not adequately visualise the valley for the number of customers paired with the fusion rate. It can be seen, from Figure 5, that the distance is relatively sensitive to the number of customers, fusion site service rate, and plasma membrane service rate. The number of customers and the fusion rate appear to have some relation such that increasing both parameters in the right ratio (such that $\frac{N}{\mu _F} \approx 18514$) is able to produce similar model outputs for a large range of both parameters. For the parameter range presented, the fit parameter values are in the region of lowest distance.

## Discussion

Insulin-dependent processes in the GLUT4 translocation pathway have previously been identified in the literature: the endocytosis rate is insensitive to insulin but exocytosis is sensitive (Satoh et al. 1993; Yang and Holman 1993; Karylowski et al. 2004; Brewer et al. 2014, 2016a), there is a flush of fusion events following insulin application (Stöckli et al. 2011; Satoh et al. 1993; Holman et al. 1994; Burchfield et al. 2013), and there is an increase in both the amount of GLUT4 recycling (Govers et al. 2004; Brewer et al. 2014; Xiong et al. 2010; Klip et al. 2019; Ray et al. 2023) and the amount of GLUT4 present on the plasma membrane (Fazakerley et al. 2022). However, the specific mechanisms that elicit these processes have remained elusive (Chadt and Al-Hasani 2020; Klip et al. 2019; Fazakerley et al. 2022). The model presented in this paper identifies a plausible mechanism by which sequestration of GLUT4 in sub-maximal insulin can occur and captures the system dynamics under insulin stimulation. The insulin dependence of a single component, namely the activity of the fusion sites, was found to be sufficient to capture the experimental observations.

Most models of GLUT4 recycling that include sequestration do not provide its specific biological mechanism. An exception is Fazakerley et al. (2022) with their proposed retention-catalyst. In this study we propose an alternative mechanism: the activity of fusion sites are insulin-dependent which causes sequestration of GLUT4 on the microtubules. The microtubules have finite capacity, providing a similar one-out one-in type mechanism to Fazakerley et al. (2022). The sequestration of GLUT4 on microtubules behind inactive fusion sites provides a reservoir readily available to mobilise with the activation of the fusion sites. Our model differs from the retention-catalyst model in that the application of insulin differentially activates the fusion machinery at the cell surface, increasing the number of pathways for GLUT4 to reach the plasma membrane. Rather than providing a slow-recycling pathway, in our model insulin initiates a quantal release from the blocked pathway (a mechanism consistent with Govers et al. 2004; Coster et al. 2004; Fazakerley et al. 2010). Significant sequestration of GLUT4 away from the recycling pathway occurs at sub-maximal (and physiological) levels of insulin (Coster et al. 2004; Romenskaia et al. 2025), with little to no sequestration at higher insulin levels.

Insulin has been observed to increase the number of fusion sites (Eyster et al. 2006; Khandani et al. 2007; Kioumourtzoglou et al. 2014). So, our queuing model, with our mechanism of insulin affecting the activity of the fusion sites, could be biologically realistic. There is not as much evidence in the literature for insulin changing the work rate of a component (corresponding to the service rate of a station in our model). Here we numerically investigated the hypothesis that the application of insulin causes an increase in the number of active fusion sites. We found this mechanism captures the overall dynamics of the experimental data.

The model was unable to completely capture all steady state levels and variance of the experimental data. This may in part be due to the scale and lack of heterogeneity in our model structures: the model uses vesicles as a proxy for GLUT4 with each vesicle assumed to carry the same amount of GLUT4, and the model has the same number of microtubules between realisations with all microtubules being the same length. It has previously been found that the insulin response of a single cell is highly reproducible, i.e., variance in data comes from the heterogeneity of responses between different cells (Burchfield et al. 2013). It is unlikely that this heterogeneity is the result of differences in insulin receptor levels (Olefsky and Kolterman 1981). Our model simulates the insulin response of a single cell. As our model is stochastic, it is possible to interpret each realisation of the model as the response of a different cell with a different predetermined fusion site activation function. The model could be extended to draw, for instance, active probabilities from a normal distribution, or to make the length of the microtubules a parameter drawn from a distribution. This would increase the variance of the model output to be more in line with the data, at the cost of extra parameters. Additional insulin-dependent processes may also help explain the discrepancy but may lead to overfitting. However, the insulin-dependent activity of the fusion sites alone can be seen to be a primary explanation for the observations.

In the current study, we have modelled all basal recycling via the microtubule pathway. Depolymerisation of microtubules impairs GLUT4 recycling and insulin-mediated GLUT4 translocation (Semiz 2003; Guilherme et al. 2000; Emoto et al. 2001; Molero et al. 2001; Fletcher et al. 2000; Karylowski et al. 2004; Dawicki-Mckenna et al. 2012). Some studies suggest that basal recycling occurs independently of the microtubules (Karylowski et al. 2004) and hence there exists a pathway from the endosome store direct to the plasma membrane. We opted not to explicitly include this pathway in the current study to minimise the number of parameters in the model. The basal flow in the current model represents the combination of both basal recycling pathways. Both basal pathways could be modelled explicitly in a future study.

Here, a scaled stochastic queuing network was used to describe the system. Further work is required to explore how network scaling influences the rate parameters, in order to better understand the relationship between scaling and the real biological rates. Further work should extend this modelling framework to include the further complexity of other processes. It has been observed that over-expression of molecular motors can overcome the blocking effects of PI3-K inhibition (Bose et al. 2004). This could indicate that the capacity of the microtubules should be further explored. In our optimised model, the capacity of the microtubules was fixed, and the system did not exhibit a flush of fusion events upon the application of insulin (as in this case the microtubules were operating at full capacity). In the model with the current architecture (number and capacity of stations) a flush of events could be induced in systems with different parameter values but at the detriment of the fit to the data. It is possible that this feature could also be incorporated in a future optimal model with different architecture. The actions at the plasma membrane could also be extended to include GLUT4 dispersal and clustering at the plasma membrane (Stenkula et al. 2010) or include more information on the signalling pathway.

## Conclusion

The closed queuing model presented in this study is a model of GLUT4 translocation dynamics using a minimal number of processes. This model tracks vesicles as they transit through four stations: endosomes, microtubules, fusion sites, and the plasma membrane. In this model, GLUT4 is sequestered on microtubules when associated fusion sites are inactive. With the optimised parameters for the scaled network, the fusion sites were found to be a rate-limiting step for the transit of GLUT4 to the plasma membrane.

We found that the activity of the fusion sites being the only insulin-dependent mechanism was sufficient to capture the dynamics observed experimentally. Using this modelling framework, we have identified a biologically plausible mechanism that reproduces the dominant features of the system and could be a primary determinant of the system dynamics.

## Supplementary Information

Below is the link to the electronic supplementary material.

Supplementary file 1 (zip 303 KB)

## Figures and Tables

**Fig. 6:**
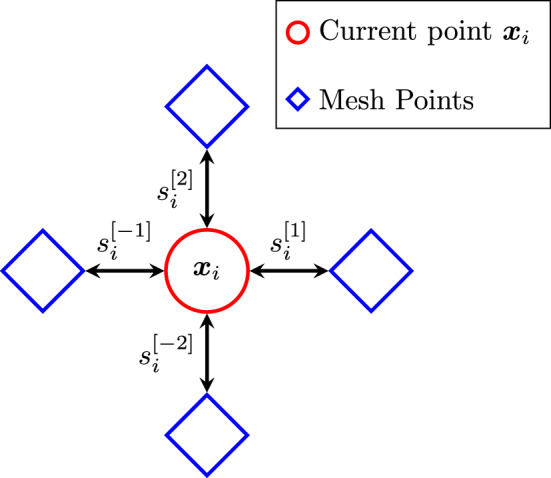
A sample of mesh points in two dimensions. Given the current point, $\varvec{x}_i$, points are calculated in along each parameter axes with the spacing of the current step size, $s_i$. Here, $s_i^{[j]}$ is the *i*th step size in the *j*th parameter dimension. On each iteration of a line search, the mesh points are used to choose a new search direction. The direction is chosen by taking the mesh point that gives the greatest decrease in the cost function from the current point, $x_i$. If no mesh points decrease the cost function then the step size is decreased

**Algorithm 1:**
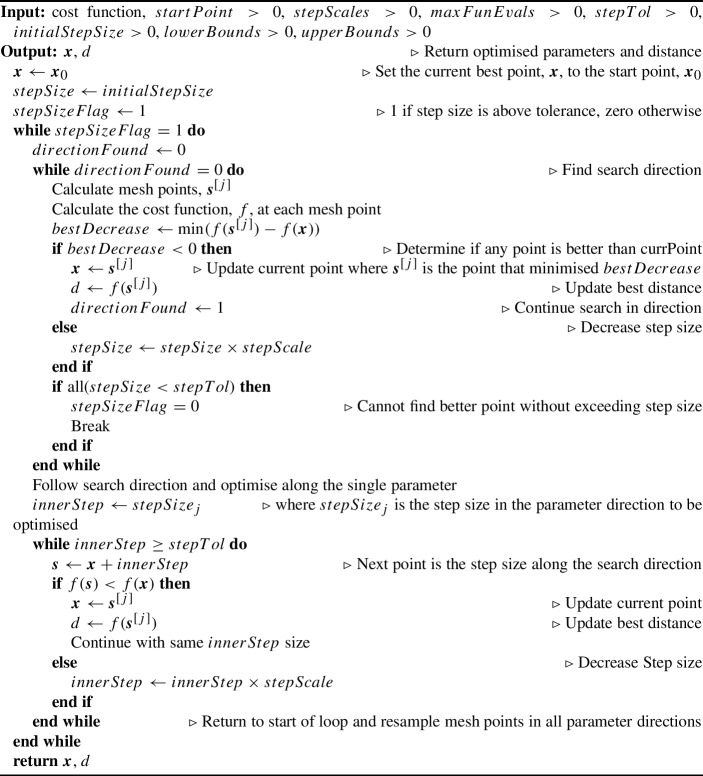
Pseudocode for fitting algorithm

## Data Availability

The code to reproduce the analyses of the paper is available in the supplementary materials.

## Line Search Method

The distance measure Sherlock et al. (2024) used here is stochastic. That is, the distance has some variance when different realisations of the model output under the same parameter set. Using this distance as a cost function (objective function), denoted as $f(\varvec{x})$, in an optimisation algorithm leads to a global optimisation problem for a stochastic (non-smooth) function. Note, that discrete derivatives of the function can be computed but this is computationally expensive. We perform a direct search where given a start point, $\varvec{x_0}$, we compute a series of points, $\varvec{x}_i$, with $i=1, 2, \dots$ where each successive point results in smaller value of the objective function, i.e., $f(\varvec{x}_{i+1}) < f(\varvec{x}_i)$.

A line search method has been used to optimise the model parameters as outlined in Algorithm 1. To attempt to reduce computation time the search direction was iteratively chosen from polling mesh points. Given initial step size, $\varvec{s}_0$, a mesh is created along each parameters axes direction of the given step size. Note, the step size is a vector with the same length as $\varvec{x}$, the vector of parameter values being optimised, so that the steps can be scaled for each individual parameters magnitude, i.e., some parameters are of the order $10^{-2}$ and others are of the order $10^4$ so equivalent step sizes are not appropriate. An example of mesh points is shown in Figure 6. An initial step size was chosen with some knowledge of the scale and sensitivity of each parameter from an initial survey of the parameter space considered. In this study, we use an initial step size of 0.1 for all parameters, except the number customers which had an initial step size of 5000. The search direction was chosen to be the direction of the mesh point that gave the greatest decrease in the cost function. If no mesh point reduced the cost function then the mesh was decreased so that the next step size is $\varvec{s}_i = \varvec{s}_0 r^{-i}$, where *r* is a factor to reduce the step size. We set $r = 2$ for this study.

After determining the next parameter direction, we minimise the cost function along that line. The step size of the mesh is maintained until the cost function no longer decreases. That is, we iterate steps $\varvec{x}_i$ in the search direction while $f(\varvec{x}_i + \alpha _k \varvec{e}_k) < f(\varvec{x}_i)$, where $\alpha _k$ is the step size in the search direction $\varvec{e}_k$. The step size is decreased in the same manner as the mesh size $\alpha _k^{[i]} = \varvec{s}_0 r^{-i}$ until the step size is smaller than the tolerance at which point the mesh is polled from that point to choose a new search direction.

The mesh size is used as the stopping criteria for the algorithm. The algorithm terminates when the mesh size in every parameter direction is less than the tolerance for that parameter direction, i.e., when $s_i^{[k]} < tol^{[k]}$ for each parameter direction *k*. Note, when individual directions step sizes are smaller than their tolerance, but the algorithm has not terminated, these mesh points are excluded from polling.
